# Supplementary figures and images for: β-asarone induces viability and angiogenesis and suppresses apoptosis of human vascular endothelial cells after ischemic stroke by upregulating vascular endothelial growth factor A
Source: PeerJ. 2024 Jun 27;12:e17534. doi: 10.7717/peerj.17534 (PMC11214739; doi:10.7717/peerj.17534)

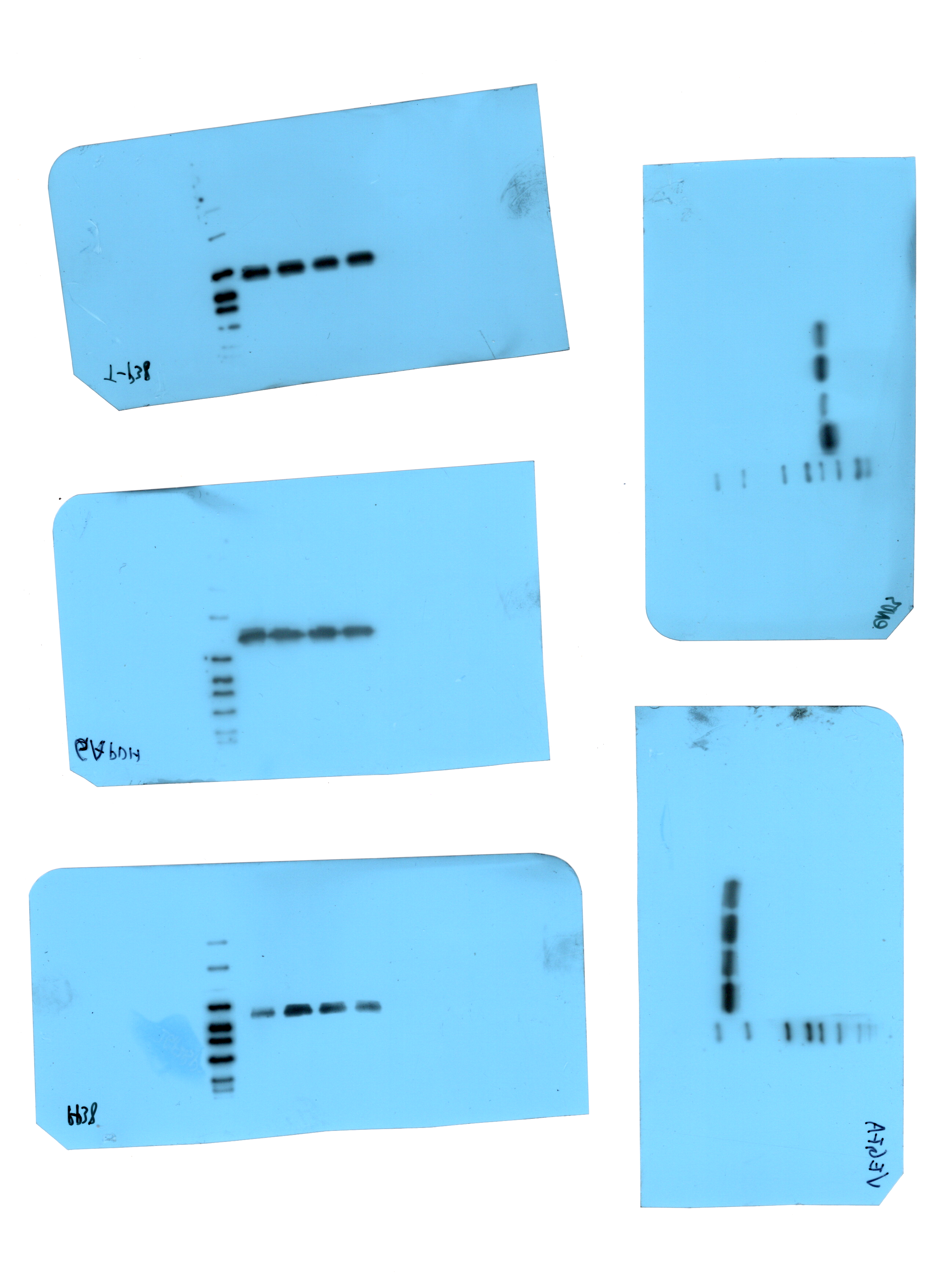

Supplement: Supplemental Information 3 [file peerj-12-17534-s003.zip › Uncropped WB blots/Figure 2B/Total.tif]

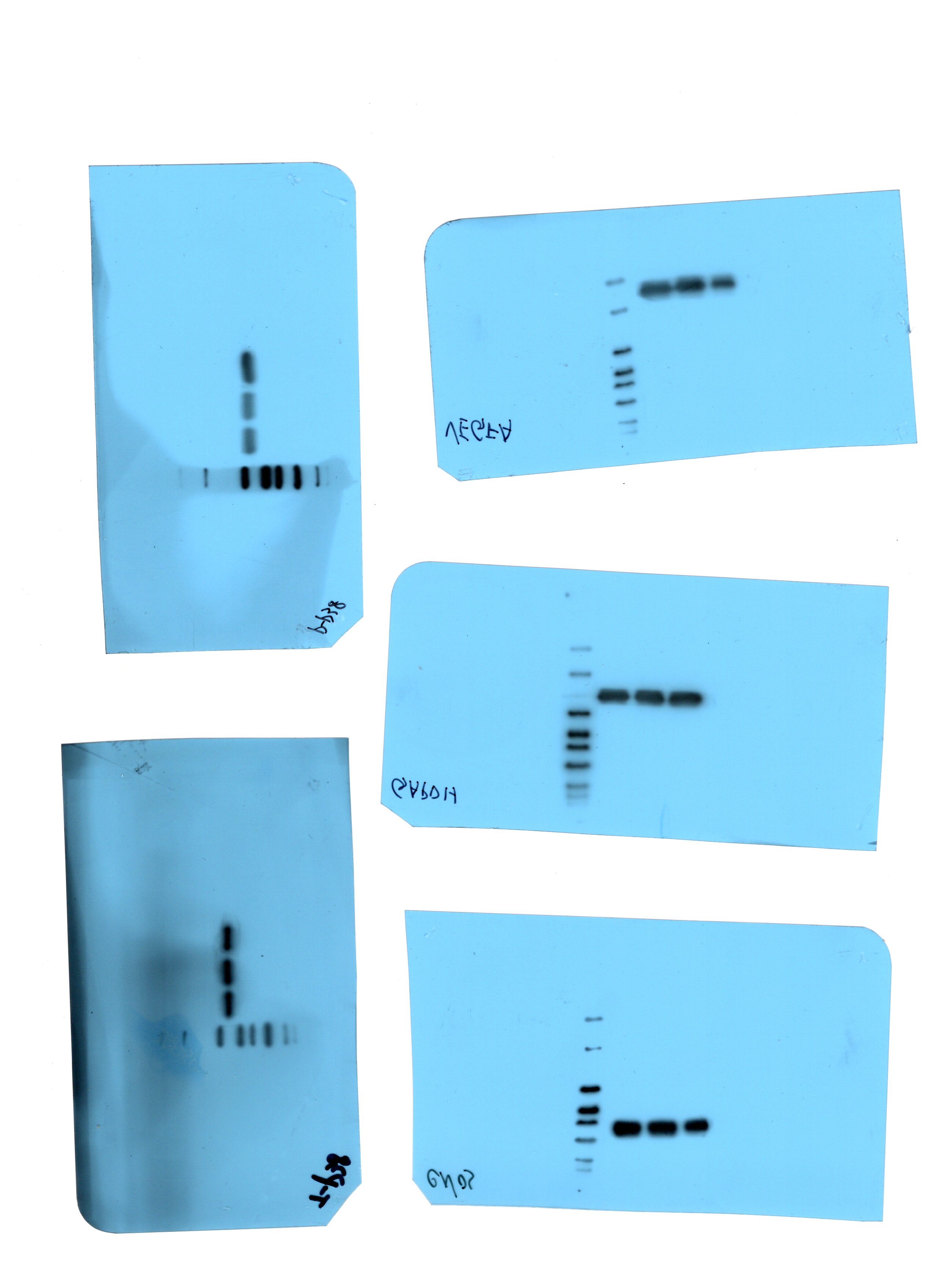

Supplement: Supplemental Information 3 [file peerj-12-17534-s003.zip › Uncropped WB blots/Figure 4B/Total.tif]

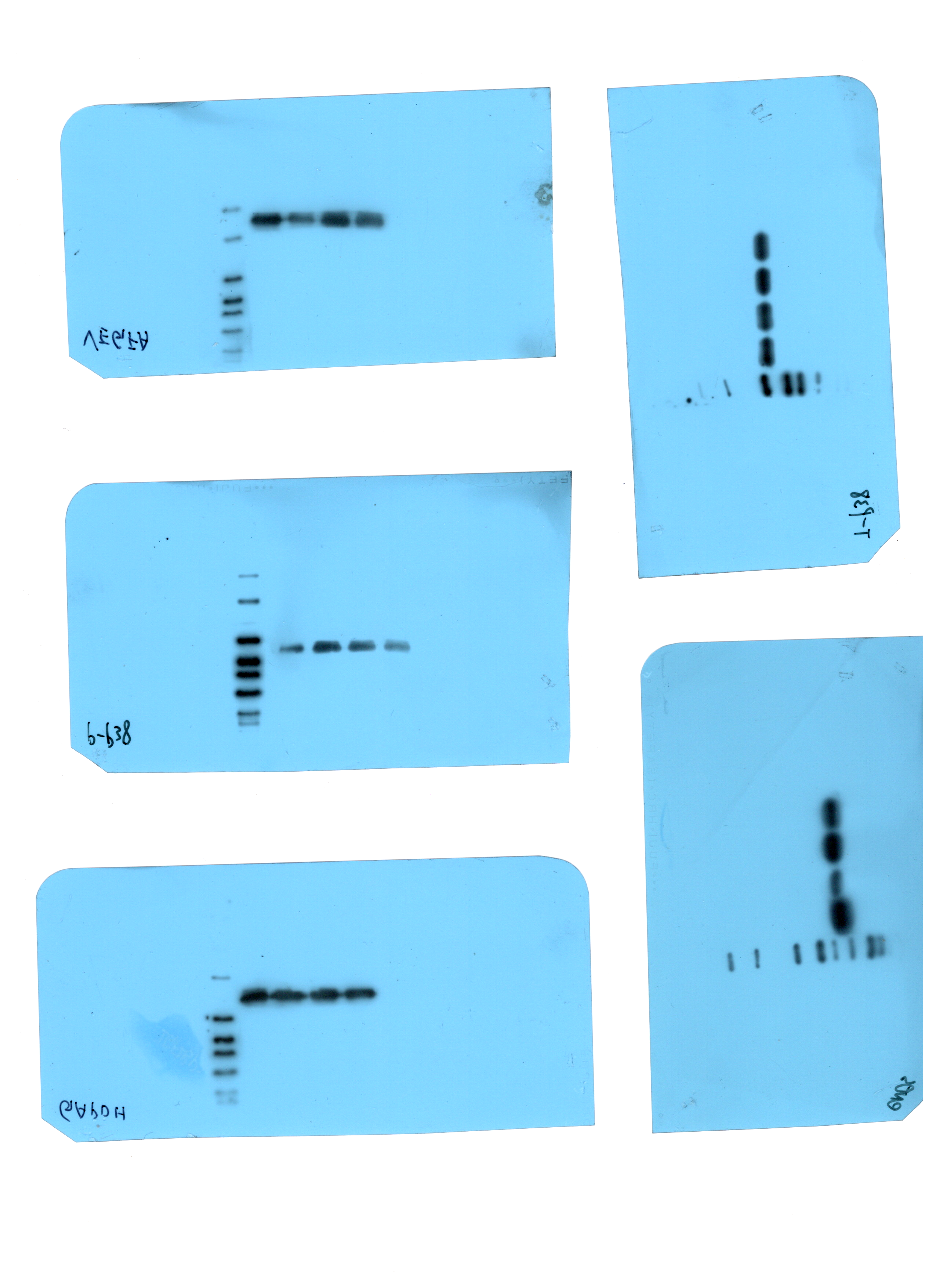

Supplement: Supplemental Information 3 [file peerj-12-17534-s003.zip › Uncropped WB blots/Figure 5E/Total.tif]

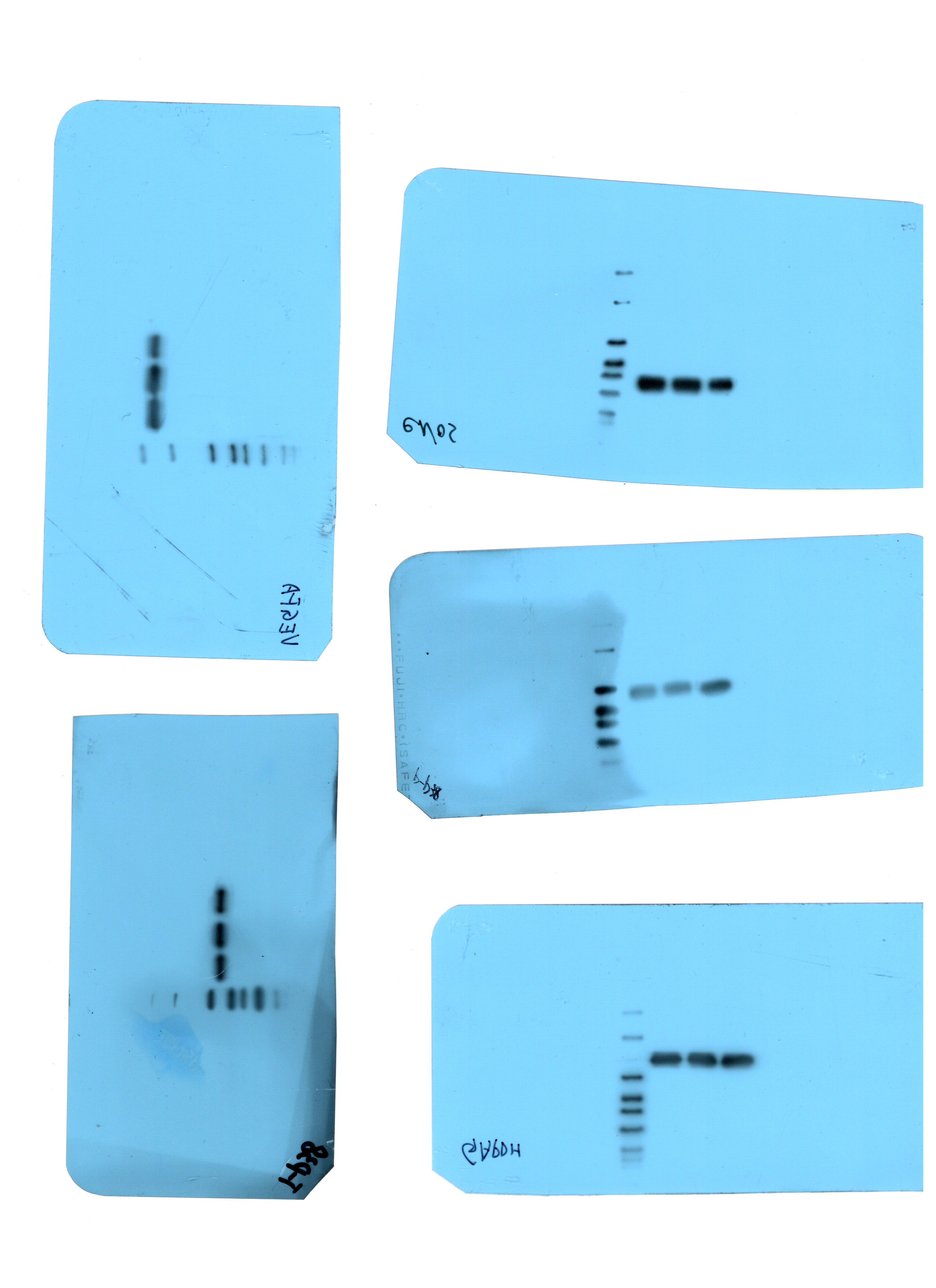

Supplement: Supplemental Information 3 [file peerj-12-17534-s003.zip › Uncropped WB blots/Figure 6A/Total.tif]
